# Comment on “Noninvasive Ventilation Weaning in Acute Hypercapnic Respiratory Failure due to COPD Exacerbation: A Real-Life Observational Study”

**DOI:** 10.1155/2019/5490510

**Published:** 2019-10-01

**Authors:** Habib Md. Reazaul Karim, Antonio M. Esquinas

**Affiliations:** ^1^Department of Anaesthesiology and Critical Care, All India Institute of Medical Sciences, Raipur 492099, India; ^2^Intensive Care Unit, Hospital Morales Meseguer, Murcia 30500, Spain

We have read with great interest the article by P. Faverio et al. published in this journal [1]. We thank the authors for being able to establish an adequate strategy of weaning from noninvasive ventilation (NIV) incorporating interruption of ventilation. The article also rightly highlights the lack of consensus and studies in this regard. Although the study has a retrospective design, the hypothesis tested and evidence provided are compelling. Yet, we consider that some interesting practical aspects are worth mentioning.

While the authors have tested the methodology of NIV interruption well, the number of days at each stage of weaning, NIV during afternoon and night or nocturnal NIV only, was at physician discretion. This is an exciting aspect and confusing as well because, eventually, the criteria may be influenced by wide individual variability, which may make it impossible to extrapolate the results. Good numbers (39%) of the patients in the chronic domiciliary NIV group were not able to be weaned by the new method and continued to be in the domiciliary NIV settings. As there are no clear criteria for such patients, it will be worth knowing the settings, whether these patients were finally stabilized, and if yes, “how.” These aspects will help readers in better application or exclusion of the new interruption-based weaning in this subgroup of patients in the future. Pneumothorax due to severe bullous emphysema is considered by the authors as a risk factor for not weaning. While we agree that such patients are prone to not weaning, pneumothorax it is more related to the selected positive inspiratory and end-expiratory pressure levels selected [2]. However, the positive pressure used by the authors was not so high, so this association was not much clear from this study. All these aspects lead us to believe that if these patients were taken into account, the results might have been different.

We again thank the authors for another interesting observation of no recurrence of AHRF during hospitalization after weaning by the new method. This is interesting and raises speculation whether this can be considered as a protective aspect of the NIV. A prospective randomized study will give us more evidence on these aspects, especially which subgroup of patients will be benefited by this new technique.

## Abbreviations

NIV: Noninvasive ventilation
AHRF: Acute hypercapnic respiratory failure.
